# Reciprocal expression of tumour-associated carbohydrate antigens (Tn/sTn) and nuclear factor-B serves as an indicator of the prognosis of oral squamous cell carcinoma

**DOI:** 10.1186/2051-1426-1-S1-P58

**Published:** 2013-11-07

**Authors:** Chi-Yu Lin, Shin Nieh, Jacqueline Whang-Peng, Jaulang Hwang

**Affiliations:** 1 Graduate Institute of Life Sciences, National Defense Medical Centre, Taipei, Taiwan; 2 Department of Pathology, National Defense Medical Centre, Taipei, Taiwan; 3 Department of Biochemistry, Taipei Medical University, Taipei, Taiwan; 4 Centre of Excellence for Cancer Research, Taipei Medical University, Taipei, Taiwan

## 

To determine whether expression of tumour-associated carbohydrate antigens (Tn/sTn) and a representative inflammation marker, nuclear factor-B (NF-B), is related to the invasiveness of oral squamous cell carcinoma (OSCC) by correlating these markers with the well-established invasive pattern grading score (IPGS) and clinicopathological parameters. Specimens from 143 OSCC patients with clinicopathological parameters were classified and scored by the IPGS system, followed by an immunohistochemical study of the expression of Tn, sTn and NF-B. Our results showed that the expression of both Tn and NF-B was significantly correlated with staging, recurrence, and distant metastasis. Both Tn and NF-B expression were positively correlated with IPGS; however, sTn expression was inversely correlated with IPGS. In addition, overexpression of Tn and NF-B was closely correlated with poor survival, whereas expression of sTn was inversely correlated with the patients’ prognosis. Our results indicate there is a reciprocal relationship between Tn and sTn expression and that these may serve as reliable indicators for evaluation of prognosis. They are also worthy of consideration as a therapeutic target for treatment of OSCC. In addition, expression of Tn rather than sTn may play an important role in late invasive OSCC via regulation of NF-B signalling.

